# Depressive symptoms among individuals identifying as asexual: a cross-sectional study

**DOI:** 10.1038/s41598-024-66900-6

**Published:** 2024-07-12

**Authors:** Sonia Lech, Monia Köppe, Maximilian Berger, Enrique Alonso-Perez, Paul Gellert, Wolfram Herrmann, Pichit Buspavanich

**Affiliations:** 1 Department of Psychiatry and Neurosciences, Charité – Universitätsmedizin Berlin, Corporate Member of Freie Universität Berlin and Humboldt-Universität zu Berlin, Charitéplatz 1, 10117 Berlin, Germany; 2 Institute for Medical Sociology and Rehabilitation Science, Charité – Universitätsmedizin Berlin, Corporate Member of Freie Universität Berlin, Humboldt-Universität zu Berlin, Berlin, Germany; 3 Research Unit Gender in Medicine, Charité – Universitätsmedizin Berlin, Corporate Member of Freie Universität Berlin and Humboldt-Universität zu Berlin, Berlin, Germany; 4 German Center for Mental Health (DZPG), Partner Site Berlin/Potsdam, Berlin, Germany; 5 Institute of General Practice and Family Medicine, Charité – Universitätsmedizin Berlin, Corporate Member of Freie Universität Berlin and Humboldt-Universität zu Berlin, Berlin, Germany; 6 Department of Psychiatry, Psychotherapy and Psychosomatics, Faculty of Health Sciences Brandenburg, Brandenburg Medical School Theodor Fontane, Neuruppin, Germany; 7 Institute of Sexology and Sexual Medicine, Charité – Universitätsmedizin Berlin, Corporate Member of Freie Universität Berlin and Humboldt-Universität zu Berlin, Berlin, Germany

**Keywords:** LGBTQIA+, Asexual identity, Depressive symptoms, Mental health, Psychology, Health care

## Abstract

Although asexuality became a growing research subject over the last decade, data on the mental health of individuals identifying as asexual is still rare. The key objective of the present study was to examine depressive symptoms among individuals identifying as asexual. Data of LGBTQIA+ (Lesbian, Gay, Bi-sexual, Trans*, Queer, Inter*, Asexual and/or + indicating others within the community) and cisgender heterosexual individuals was collected through an online survey during the COVID-19 lockdowns in Germany. The survey included questions about sexual and gender identity, depressive symptoms, and asexual identity. An analysis of N = 6601 participants was conducted. A total of n = 445 individuals identified as asexual. Regression results indicated identifying as asexual being significantly associated with higher depressive symptoms. Results suggest that individuals identifying as asexual represent a vulnerable group within the group of sexual minorities, one that fundamentally requires special psychosocial support, especially in times of pandemics.

## Introduction

The Asexual Visibility and Education Network, a network and archive of resources on asexuality, defines asexual individuals as individuals who “*are not drawn to people sexually and do not desire to act upon attraction to others in a sexual way*”^1^. Asexuality was long considered a sexual desire disorder, but the definition has changed over time. It is now understood to be a sexual orientation. Following a literature review asexuality was defined as “*a heterogeneous entity that likely meets conditions for a sexual orientation, and that researchers should further explore evidence for such a categorization*”^2^. The asexual community includes a spectrum of identities, including grayasexual describing individuals who rarely or only under specific circumstances experience sexual attraction and demisexual describing individuals who experience sexual attraction only after an emotional connection^3^. More community-centered definitions of asexuality emphasize that it is an umbrella term that exists on a spectrum The spectrum ranges from people who feel no sexual attraction at all (“asexual”) to people who can feel basic sexual attraction (“allosexual”, sometimes also “zedsexual”). The spelling using an underscore ”_” (“A_sexuality”) or the term “Aspec” is sometimes used to express this spectrum. Individuals identifying as asexual often have little interest in sexual intercourse, but can desire intimate emotional relationships. However, asexuality is not usually associated with aromanticism. The lifestyles and love styles of individuals identifying as asexual are diverse: *“Some don't like physical contact at all, others like kissing and cuddling. Some masturbate, some have a sense of eroticism or a fetish. Some find sexual intercourse disgusting, others are completely indifferent to it and a few are keen to experiment. What they all have in common is that they don't see sexual intercourse as a necessary expression of love*.”^4^. Therefore, the term asexuality includes a variety of non-sexual attractions and at the same time does not exclude people on this spectrum from performing sexual intercourse.

When conceptualizing the term, it is important to consider that the spectrum of asexuality has been erased and denigrated in our society^5^. Hammack, Frost and Hughes emphasize the normative assumptions about intimacy “that privilege heterosexual monogamy and the biological family unit, presume binary cisgender identities, essentialize binary sexual identities, and view sexual or romantic desire as necessary.”^6^. Instead they propose a queer paradigm to study relationship diversity. When defining asexual identity, allonormativity and heteronormativity must be taken into account.

While asexuality became a growing subject during the last decade (health) data on individuals identifying as asexual remains rare. The definition and measures of asexuality vary widely across research leading to a wide range of estimates of prevalence across studies^7^. For example, in a UK sample a study reported estimates of people identifying as asexual ranging from 0.6 to 5.5%^8^. Results from large-scale national probability studies of British residents reported estimates between approximately 0.4–1%^8–10^. A study from Finland reported that 3.3% of women never felt sexually attracted to anyone^11^: Overall, there is a higher proportion of women who identify as asexual, compared to men^3,12^. Furthermore, asexual individuals are found to be less likely in a romantic relationship^3,10,12^. In this context the term *allosexual* was established by the asexual community to describe everyone who experiences sexual desire towards others—anyone who is not asexual^1^. Moreover, studies including asexual individuals often exclude those individuals from the main analysis^13^ or categorize individuals identifying as asexual into “other groups”^14,15^. This study aims to address this gap by examining a substantial sample size of asexual individuals as part of the main research question.

In addition to demographics, recent research has been focusing on the link between asexuality, well-being and (mental) health. There is evidence indicating specifically higher rates of depressive symptoms, anxiety, suicidality and psychoticism among asexual men, compared to heterosexual men^16^. Further, a recent study found that 64.8% of individuals identifying as asexual reported experiencing minority stress based on sexual and/or romantic orientation^17^. In the same study, about 32% of those individuals reported suicidal ideation, about 11% had suicide plans, and about 3% attempted suicide in the past 12 months. Previous empirical evidence indicates an association between asexuality and higher rates of mood and anxiety disorders^16,18^.

Conceptual frameworks, such as the Minority Stress Model, explain the link between discrimination, social stress, and mental health among LGBTQIA+ communities (Lesbian, Gay, Bi-sexual, Trans*, Queer, Inter*, Asexual and/or others within the community)^19,20^. The model postulates that belonging to sexual minority groups entails a stress exposure, which negatively affects well-being^21^. These stressors include stigmatization, discrimination or exposure to violence. Overall, recent studies conducted during the COVID-19 pandemic reported poorer mental health among individuals from minoritized sexual identities^15,22^. However, asexual and bisexual individuals are particularly vulnerable to poorer well-being^22,23^. A multivariate meta-analysis found that asexual and bisexual individuals were found at greater risk of higher levels of depressive symptoms compared to gay/ lesbian and heterosexual individuals^24^. Asexual individuals reported higher internalized LGBTQIA+ -phobia and poorer mental health compared to allosexual individuals^25^.

However, the link between asexuality and mental health remain inconsistent, as other studies have not found a link between asexuality and poorer mental health^12,26^. For example, Rothblum et al. found no differences between asexual individuals and allosexual individuals regarding general well-being, life satisfaction, and social support^27^. Nevertheless, until now, there has been a notable absence of in-depth analysis concerning the mental health of asexual individuals. Further research is needed to gain a better understanding of the association between the spectrum of asexuality and mental health. Moreover, many studies investigating the mental health of asexual individuals have limited their analysis to binary gender options (male and female), neglecting other gender identities^16,26,28–30^. This highlights a significant gap in the specific analysis of the mental health of asexual individuals.

### Aims of the study

The study aims to address the paucity of research about asexual individuals by focusing on this population and examining their mental health, specifically depressive symptoms, in comparison to allosexual individuals. The study has two primary objectives:

1. To provide descriptive data on asexual individuals in Germany, which is currently lacking in the literature. While the study does not aim to assess changes in depressive symptoms before and during the COVID-19 pandemic through a before-and-after comparison, it acknowledges the pandemic as a significant influence on the study environment.
2. To investigate potential differences in depressive symptoms between asexual and allosexual individuals. Based on prior research indicating higher levels of mental health problems among asexual individuals, we hypothesize that those identifying as asexual will exhibit greater depressive symptoms compared to allosexual individuals.

We will first conduct comparisons of age, sexual orientation, and gender identity between asexual and allosexual individuals. This will provide a foundation for understanding the demographic characteristics of the asexual population in our study. Subsequently, we will examine the differences in depressive symptoms between the two groups, with the expectation that asexual individuals will report higher levels of depressive symptoms compared to their allosexual counterparts.

## Material and methods

### Participants and procedure

Cross-sectional data was collected using an online survey during the first (March/April 2020) and the second (January/February 2021) COVID-19 lockdowns in Germany, with data acquisition being open each time for two respectively 3 weeks. To oversample LGBTQIA+, LGBTQIA+ groups and organizations in Germany were contacted and asked to distribute the survey and share the survey on their social media. Calls for participation were announced on social media feeds that specifically engage with the LGBTQIA+ population.

The study was conducted in accordance with the Declaration of Helsinki. Data collection was anonymous, and all participants gave informed consent prior to participation. As data acquisition was anonymous and without identifying variables, no ethical approval was necessary from the ethics committee of the Charité – Universitätsmedizin Berlin. Participants did not receive any kind of incentive or remuneration.

### Measures

*Age* was assessed using an ordinal scale, where participants could sort themselves in the following age ranges: “18–25 years”, “26–35 years”, “36–45 years”, “46–55 years”, “56–65 years”, “66 to 75 years” and “76 years or older”. For the purpose of the main research question the categories “46 to 55 years”, “56 to 65 years”, “66 to 75 years” and “76 years or older” were summarized into one category “46 years or older”.

#### Sexual orientation

Sexual orientation was assessed through a multiple answer questions featuring the orientations: “aromantic”, “asexual”, “bisexual”, “gay [German slang for “schwul”]”, “heterosexual”, “homosexual”, “lesbian”, “queer”, “pansexual” and “alternative”. Every orientation could be chosen and multiple answers were possible. The categories aromantic and queer were only added in the second wave of the survey. For further analysis, the dichotomous variable asexual identification (yes = asexual identity, no = allosexual identity) was coded. Further, sexual identity was created as a dichotomous variable: heterosexual versus queer (bisexual and/or gay and/or homosexual and/or lesbian and/or queer and/or pansexual).

#### Gender identity

Gender identity was assessed with the same multiple answer sheet offering the identities: “cis”, “inter”, “man”, “non-binary”, queer”, “trans”, “woman”, and “alternative”. Each category could be chosen and multiple answers were possible. For further analysis, the dichotomous variable gender identity: TIN (trans* and/or inter and/or non-binary and/or queer) yes or no.

#### Depressive symptoms

Depressive symptoms were measured with the 8-item questionnaire “Depressive symptoms in a non-clinical context”^31^. The items measure impairments of well-being on a 7-point Likert scale with 1 indicating no agreement and 7 indicating strong agreement. The scale was calculated as a mean of the 8-items, with higher scores indicating higher levels of depressive symptoms. Reliability was high with *Cronbach’s alpha* of 0.90.

### Analysis

First, descriptive analyses (i.e., frequencies for categorical variables) for all participants across both cohorts were calculated. Next, a standardized comparison of means was performed with a chi-square for categorical variables. Third, to test the main research question, a multiple regression with depressive symptoms as a dependent variable was performed. Age categories (18–25 years old, 26–35 years old, 36–45 years old, and 46 years or older), gender identity (TIN yes or no), and sexual orientation (heterosexual yes or no) as well as asexual identity (yes or no) were included in the model. Only participants were included in the analysis that provided information on asexual identity. Missing data was excluded in the analysis. For the regression, adjusted R2, F values (F), degrees of freedom and significance levels (p) are reported. All tests of significance were based on a *p* < 0.05 level and confidence interval (CI) of 95%. All statistical analyses were performed using IBM SPSS Statistics for Windows V.27.0.

### Statement of ethics

All participants gave their informed consent before taking part in the online survey. Data acquisition was anonymous without identifying variables. Because of the anonymity of the study, the consultation of an ethical review board was not necessary. Participants did not receive any kind of incentive or remuneration.

## Results

### Participants

A total of N = 6870 people participated in the survey. Data of N = 6601 participants were included in the analysis. A total of n = 445 (6.7%) participants of the sample reported an asexual identity. Distributions and comparisons of age, sexual orientation, and gender identity for all individuals and between individuals identifying as asexual or allosexual can be obtained from Table 1. Overall, about 42% (n = 186) of individuals identifying as asexual were in the age group 18–25 years old and 44% of individuals identifying as asexual (n = 196) identified as female. One third (33.8%) of individuals identifying as asexual identified as queer, lesbian, or gay. Individuals identifying as asexual were more likely to report a TIN gender identity compared to allosexual individuals (58.8% versus 18.2%).

**Table 1:** Characteristics of and comparisons between asexual and allosexual individuals.

	All participants (N = 6601)		Allosexual (N = 6156)		Asexual (N = 445)		*χ2 (df)*	*p*-value
	*n*	*%*	*n*	*%*	*n*	*%*		
Age								
18–25 Years	1426	21.8	1240	20.3	186	41.8	111.95	< 0.001
26–35 Years	2151	32.9	1989	32.6	162	36.4	2.67	0.102
36–45 ears	1450	22.2	1397	22.9	53	11.9	29.14	< 0.001
≥ 46 ears	1513	23.1	1469	24.1	44	9.9	47.12	< 0.001
Sexual orientation								
Aromantic	118	2.9	40	1.1	78	26.0	606.52 (1)	< 0.001
Bisexual	969	14.7	904	14.7	65	14.6	.002 (1)	0.964
Gay	1521	23.0	1487	24.2	34	7.6	63.83 (1)	< 0.001
Heterosexual	1047	15.9	1022	16.6	25	5.6	37.52 (1)	< 0.001
Lesbian	1143	17.3	1084	17.6	59	13.3	5.49 (1)	0.019
Pansexual	737	11.2	663	10.8	74	16.6	14.34 (1)	< 0.001
Queer	949	23.6	849	22.8	100	33.3	17.17 (1)	< 0.001
Homosexual	2321	35.2	2280	37.0	41	9.2	140.92 (1)	< 0.001
Gender identity								
Cisgender	1661	25.2	1533	24.9	128	28.8	3.29 (1)	0.070
Inter*	39	0.6	29	0.5	10	2.2	22.30 (1)	< 0.001
Men	2521	38.2	2431	39.5	90	20.2	65.25 (1)	< 0.001
Non-binary	737	11.2	578	9.4	159	35.7	290.32 (1)	< 0.001
Trans*	601	9.1	508	8.3	93	20.9	80.21 (1)	< 0.001
Women	2388	36.2	2192	35.6	196	44.0	12.80 (1)	< 0.001
Queer	841	20.9	753	20.2	88	29.3	14.01 (1)	< 0.001

Only participants were included in the analysis that provided information on their asexual or allosexuell (not asexual) identity. χ2 = Pearson Chi-Square, *df* = degrees of freedom.

### Depressive symptoms

Results of the multiple regression model are presented in Table 2. The regression model explained X% of the variance in depressive symptoms (adjusted *R*^2^ = 0.117, *F*(6,4476) = 0.569, *p* < 0.001). Further, identifying as asexual was, over and above all other independent variables, significantly associated with higher depressive symptoms (*β* = 0.059, *CI* = 0.165, 0.5551, *p* < 0.001).

**Table 2:** Results of the multiple regression models for depressive symptoms.

	Depressive symptoms					
	Estimate	SE	95% CI		ß	adjusted R^2^
			*LL*	*UL*		
						0.117***
Constant	3.118***	0.090	2.942	3.294		
Age						
18—25 years	0.831***	0.053	0.728	0.934	0.279	
26 – 35 years	0.492***	0.048	0.399	0.586	0.187	
36 – 45 years	0.217***	0.053	0.113	0.320	0.071	
over 46 years (reference)	–	–	–	–	–	
Gender identity (TIN)	0.474***	0.050	0.376	0.572	0.138	
Sexual orientation (heterosexual)	− 0.413***	0.044	− 0.500	− 0.325	− 0.132	
Asexual identity (yes)	0.376***	0.084	0.211	0.541	0.064	

*Estimate*, Unstandardized Estimate; *SE*, Standard error; CI, confidence interval; LL, lower limit; UL, upper limit; *β* = Standardized Beta, TIN = Trans*, Inter*, Non-binary; *p* < 0.05, **p < 0.01, ***p < 0.001.

## Conclusion

The key objectives of this study were to examine spectrum of asexuality and to study depressive symptoms among individuals identifying as asexual compared to allosexual individuals in Germany. The data were collected during the initial phases of the COVID-19 pandemic lockdowns. Our findings indicate differences between age, sexual orientation and gender identity between asexual individuals and allosexual individuals. Individuals identifying as asexual reported higher levels of depressive symptoms.

A total of 445 individuals identifying as asexual took part in the survey, comprising 6.5% of the sample. This proportion exceeds the prevalence rate between 1 and 5. 5% reported in previous studies^8,32^. It is essential to note that the overrepresentation of LGBTQIA+ community members in our study and the self-identification of asexuality without further elaboration may have contributed to the higher frequencies observed. Consistent with prior research, the data collected in this study demonstrates substantial associations between individuals identifying as asexual and non-binary and trans* identities^12,27,33,34^. Furthermore, a notable predominance of female gender identity among individuals identifying as asexual, consistent with previous research was evident^35^.

Looking at depressive symptoms, individuals identifying as asexual reported higher depressive symptoms in comparison to the allosexual group during the pandemic. Similar to previous work studying a potential vulnerability of sexual and gender minorities with regard to (mental) health individuals identifying as asexual reported higher levels of depressive symptoms^14,15,36^. This is independent of their sexual orientation and gender identity. The present finding is also in line with past research examining mental health among individuals identifying as asexual^22,23^. The increased harmful stereotypes about asexuality, also from within the LGBTQIA+ Community, reported in previous studies may increase the mental health difficulties in individuals identifying as asexual and be a reason for this effect^16,37^. Future research is needed in order to better understand the mechanisms behind the notable vulnerability of individuals identifying as asexual for greater mental health burden.

### Strengths and limitations

A particular strength of this study was the high number of participants. Further, we included a vast range of sexual and gender identities, to foster inclusivity in examining mental health among LGBTQIA + individuals. Due to the various selection possibilities, a more detailed analysis was possible in comparison to studies that for example assess gender in a binary format^26,28^. The multiplicity of gender identities recorded and the large sample size have produced new insights about asexuality.

Nevertheless, a few important limitations must be outlined. The main limitation of the present study is its cross-sectional design. The study was recruited during the COVID-19 pandemic without longitudinal data before or after the pandemic. Therefore, we cannot conclude whether the pandemic or other relevant factors related to this issue might have influenced our results. Another limitation is the lack of data on education and income. Intersectional approaches when studying mental health at the intersection of gender identity, sexual identity, income/class and race/ethnicity are of great importance. These factors intersect to jointly impact mental health in complex ways, and where not considered in the present analysis. For example, a recent study reported higher major depression and generalized anxiety rates among low-income students, students of color and LGBTQIA+ students, including asexual individuals^38^. Same-sex parents and single LGBTQIA+ parents also had a higher risk of poverty during the pandemic^39^. The present analysis did not record education, income, or race. Future research should include other domains of diversity such as income and ethnicity should be considered in further studies when examining mental health of sexual minorities^40^. Further, the lack of differentiating between the sexual and the romantic orientation in the present analysis represents another limitation. When studying the spectrum of asexuality, it is of great importance to differentiate between the sexual and the romantic orientation. Although past research found, that asexual individuals are less likely than allosexual individuals to be a romantic relationship^12^, individuals identifying as asexual can experience romantic attraction^3^. Asexual communities often make a distinction between sexual and romantic attraction. Romantic attractions include labels like heteroromantic, homoromantic, and biromantic. However, since the present study did not only focus on individuals identifying as asexual, it was challenging to capture romantic and sexual orientation separately, a problem that has been already reported previously^41^. However, future research should include a distinction between the sexual and romantic orientation in their survey when studying the asexual individuals. Hopefully, with increasing acceptance and visibility of the asexual community, these formulations will also become more common and discourses around desire more inclusive. Additionally, the self-identification of asexuality did not allow for placing the individuals on a spectrum but just ticking a box. Thus, e.g., demisexuality as option was missing. Finally, as already mentioned, the oversampling of the present study needs to be addressed as a limitation. The sample of the presents study is not representative for the general population in Germany. Present data was collected in Germany, a country with equality laws and policies. Past research has numerous times outlined differences in equality for LGBTQIA+ individuals across countries^42^. Thus, present results are mainly transferable to western countries^9,43^.

## Conclusion

In conclusion, this study highlights the need for further research into the spectrum of asexuality, including an exploration of social support among individuals identifying as asexual. Health practitioners, as well as members of the LGBTQIA+ community, should be better informed about the asexual spectrum to support the specific needs of individuals identifying as asexual and combat harmful stereotypes. In particularly (mental) health practitioners, which in recent studies were found to be part of a stigmatizing experience, should be more informed about the spectrum of asexuality and know how to assess the specific needs of individuals identifying as asexual^44,45^. Our findings suggest that knowing the sexual orientation identity of their patients may be helpful for health professionals to make the best diagnostic and treatment decisions, which should be demonstrated in further studies emphasizing community-based approaches and tailored interventions.

## Data Availability

Data is stored in a non-publicly available repository. However, data are available from the corresponding author on request.
